# Evaluation of Cytotoxicity and Mould Contamination of Selected Plants from Meadows Covered by the Agri-Environmental Program

**DOI:** 10.3390/toxins11040228

**Published:** 2019-04-17

**Authors:** Magdalena Twarużek, Romuald Dembek, Dariusz Pańka, Ewelina Soszczyńska, Ewa Zastempowska, Jan Grajewski

**Affiliations:** 1 Department of Physiology and Toxicology, Institute of Experimental Biology, Faculty of Natural Sciences, Kazimierz Wielki University, Chodkiewicza 30, 85-064 Bydgoszcz, Poland; twarmag@ukw.edu.pl (M.T.); eweso@ukw.edu.pl (E.S.); jangra@ukw.edu.pl (J.G.); 2 Department of Agrotechnology, Faculty of Agriculture and Biotechnology, UTP University of Science and Technology, Kaliskiego 7, 85-796 Bydgoszcz, Poland; dembekro@utp.edu.pl; 3 Department of Phytopathology and Molecular Mycology, Faculty of Agriculture and Biotechnology, UTP University of Science and Technology, Kordeckiego 20, 85-225 Bydgoszcz, Poland; dariusz.panka@utp.edu.pl

**Keywords:** meadow plants, cytotoxicity, fungal contamination, endophytes

## Abstract

The aim of the study was the evaluation of selected species of meadow plants obtained from the first cut from the area covered by the agri-environmental program ‘Natura 2000’ in terms of the presence of cytotoxic compounds detected by the MTT test and the level of fungal contamination. The research was carried out on plant species that were evaluated differently in previously used methods for quality assessment of pasture feeds according to Klapp and Filipek. Twenty-six plant species were harvested in 2014 from meadows located in the valley of the Bydgoszcz Canal. Mycological examination of meadow plant samples was carried out according to PN-ISO 7954:1999. Cytotoxicity evaluation was performed using the MTT [3-(4,5-Dimethylthiazol-2-yl)-2,5-diphenyltetrazolium bromide] test. Selected samples were also subjected to evaluation of the endophytes occurrence in grasses using PCR. Natural meadow positions included in the study were dominated by moulds belonging to *Humicola* spp., *Alternaria* spp., *Cladosporium* spp., *Torula* spp., *Fusarium* spp. and *Mucor* spp. The highest level of fungal contamination was observed for *Carex acutiformis* Ehrh. The most infested grasses were *Deschampsia caespitosa* (L.) P.Beauv., *Festuca arundinacea* Schreb. and *Lolium perenne* L. The MTT test showed that the most cytotoxic species were *Arrhenatherum elatius* (L.) P.Beauv. (IC_50_ 1.563 mg/mL) and *Ranunculus repens* L. (IC_50_ 3.125 mg/mL). *Epichloë* endophytes were detected in one of 13 examined grass samples. Our own research suggests that previously used feed quality assessments should be verified by introducing modern methods of molecular biology and instrumental analysis. Results of this study may broaden the knowledge of the causes of problems resulting from feeding of roughage, mainly from natural meadows, and help in creating new rankings of the feed value of meadow sward components.

## 1. Introduction

Grassy ecosystems are of great natural importance due to their phytosanitary, structure-forming and anti-erosion functions. By absorbing significant amounts of air pollutants, these communities play the role of the so-called ‘green nature filter’. Regardless of the aforementioned functions, grasslands are primarily a basic source of high-value feed in the form of green fodder, hay and silage [1]. Economically, grasses play a major role among fodder plants necessary for the production of meat and milk. Under European conditions, grasslands are the basic forage area for ruminants. Grasses and legumes grown on them were, and will undoubtedly be, the basis for feeding ruminants in Poland, Europe and on other continents. Hence, grasslands are the best source of the cheapest and most highly valuable feed for cattle.

For the majority of ruminants, especially cattle, feeding criteria are met by sward (characterized by high density) composed of grass (60–70%) and legume plants (10–30%) with a small proportion of herbs, which provide flavour, while slightly improving the yield. Grasses determine the yield and energy value of the pasture sward. Despite the presence of many grasses on pastures, the most valuable and recommended for use in pasture mixes are: *Lolium perenne* L., *Poa pratensis* L., *Dactylis glomerata* L., *Festuca pratensis* Huds., *Phleum pratense* L., *Agrostis gigantea* Roth, *Festuca rubra* L. (creeping form). Among legumes, this is *Trifolium repens* L. [2]. However, in Polish conditions, the latent production potential of meadows and pastures is often unused, since grasslands are usually treated without much care, which leads to a reduction in their production efficiency [1].

The nutritional value of roughage depends on many factors associated with species characteristics modified by habitat and anthropogenic factors, mainly fertilization and harvesting dates. Interrelations cause high variability, however, the basic principles of factor interactions are known, e.g., given by Falkowski [3] and Rutkowska [4] and, based on simple chemical analyses, it is possible to verify and estimate the quality of feed from grassland. In meadow sward, especially in natural meadows, there are many species of varying nutritional value, from very good to bad, and even ones considered harmful, reducing the feed intake or acting adversely on animal organisms. This knowledge results from many years of observation and research in the field of animal nutrition, mainly in ruminants.

At present, in the Polish meadow practice, the comprehensive, although simplified, valorization of feed value is carried out using the 14 grade scale of use-value numbers proposed by Filipek [5]. Previously, a 10 point Klapp scale was used [6]. Both methods of synthetic assessment of sward value were based on the knowledge of typical nutritional parameters, as well as the concentration of harmful compounds that reduce the effects of nutrition or even threaten animal health. Both classifications are over 45 and 60 years old, respectively. Based on newer research methods, there is a need to verify the quality of species that are part of meadow communities and roughage made of them. The above classifications were useful when mainly hay was obtained from the meadow sward of the first or second cut, and animals could omit lower-quality species. Currently, on the meadow communities covered by the agri-environmental program, the first cut is much overgrown and has worse microbiological status. Verification of species quality by the Klapp or Filipek method with a much-delayed harvest is less useful, because the first cut is used for silage or haylage, which then in feed carts with the use of raw materials is a complete feed TMR (Total Mixed Ration). A more complete assessment of the quality of green fodder can be obtained by assessing the degree of mould infestation, their cytotoxicity and the level of secondary metabolites—mycotoxins using modern instrumental methods. The ‘Natura 2000’ initiative required a change in the management of meadows. The delayed harvest of meadow sward of the first cut under moist atmospheric conditions is conducive to infection of plants by a number of species of fungal flora. A large proportion may be pathogenic mould fungi, the secondary metabolites of which exhibit toxicity to human, animal or plant organisms. Mycotoxins have a wide range of action and toxic effects, from chronic contamination to acute poisoning. They can also appear as masked mycotoxins. Chemical transformation is often done by plants by coupling with another molecule, often glucose. The conjugated mycotoxins therefore remain active, because they can be released by hydrolysis in the digestive tract or fermentation process and return to initial toxicity. Numerous factors affect the development of fungi and the production of mycotoxins. One fungus can produce various mycotoxins and one mycotoxin can be produced by many fungal species. In raw materials and plant feeds, the most common mycotoxins are: trichothecenes type A and B, zearalenone, fumonisins, ergot alkaloids, enniatins, mycotoxins from *Alternaria*, *Mucor*, *Monascus* and less frequently, ochratoxin and aflatoxin [7,8]. Some specific anti-nutritional compounds are produced by grasses [9] and plants from other botanical families [3]. According to the literature, some of the better recognized ones are alkaloids. In the Poaceae family, about 20 alkaloids have been identified, 130 alkaloids in the Liliaceae family, and in the Fabaceae even 150. Their harmfulness is not the same, but all cause cirrhosis, various chronic inflammations, and also act neurotoxically. The cyanogenic glycosides found in about 2000 species from 110 botanical families also have a harmful effect. Phytotoxic components also include coumarins commonly found in species from the Poaceae, Asteraceae, Fabaceae and Apiaceae families. The harmfulness of all specific compounds is to a large extent a derivative of their concentration in plants [3,9].

More and more attention is also paid to anti-nutritive substances produced by fungal endophytes from the genus *Epichloë* (previously classified also to the genus *Neotyphodium*), commonly found in numerous species, including fodder species of the *Lolium* and *Festuca* genera [10,11]. The endophytes may be used in biological plant protection, as they can make grass resistant to pests, nematodes or some diseases, and at the same time, pose threat to the health of herbivorous animals, through the presence of ergovaline metabolites and lolitrem B [12,13,14].

More and more information on the occurrence of specific anti-nutritional compounds indicates the need to verify existing criteria used to evaluate the beneficial or harmful properties of meadow sward components. So far, there have been no studies on fungal infestation and assessment of the cytotoxicity of meadow sward from areas with delayed harvest of the first cut, included in the ‘Natura 2000’ program.

In this light, current knowledge of the dietary value of meadow plants requires verification, primarily with regard to the concentration of anti-dietary compounds as well as the plants’ susceptibility to fungal pathogens. Mycotoxins and phytotoxins can have a wide range of mechanisms of action and toxic effects. Most of the phytotoxins undergo enzymatic biotransformation in the gastrointestinal tract, mainly in liver and kidneys, to specific, less toxic metabolites. However, in ruminants, especially hungry, their hydrolysis already occurs in the rumen and clinical symptoms can be immediate. The main phytotoxins are cyanogenous glycosides, toxic amines, isoquinoline alkaloids, phenols, saponins, solanins and triterpenes. There are also a number of poisonings with plants; the toxic substances of which have not yet been identified (blood). In the case of mycotoxins, the scope of their decontamination is much more difficult. The research essentially includes in vivo fermentation processes with inoculums or in vitro studies upon rumen microorganisms. It is erroneous that silage reduces mycotoxins. Most certainly, fungal flora dies in this process. It was found that only some fungal toxins are transformed into less toxic metabolites or conjugated derivatives in the digestive tract. This process partly occurs in the rumen and, among others, applies to type B trichothecenes and ochratoxin A. In contrast, zearalenone (ZEN), the main metabolite of Fusarium, is converted by microorganisms to 10 fold more toxic metabolites α- and β-zearalenol (ZOL). The quoted data indicate that toxic products of mould fungal metabolism simultaneously reduce rumen microflora, which decreases the animal’s organism performance [7,15].

The aim of the study was the evaluation of selected species included in the Klapp [6] and Filipek [5] classification in the range from least to most harmful, in terms of the presence of cytotoxic compounds detected by the MTT test and the level of fungal contamination.

## 2. Results

### 2.1. Fungal Contamination of Meadows

**Figure 1:**
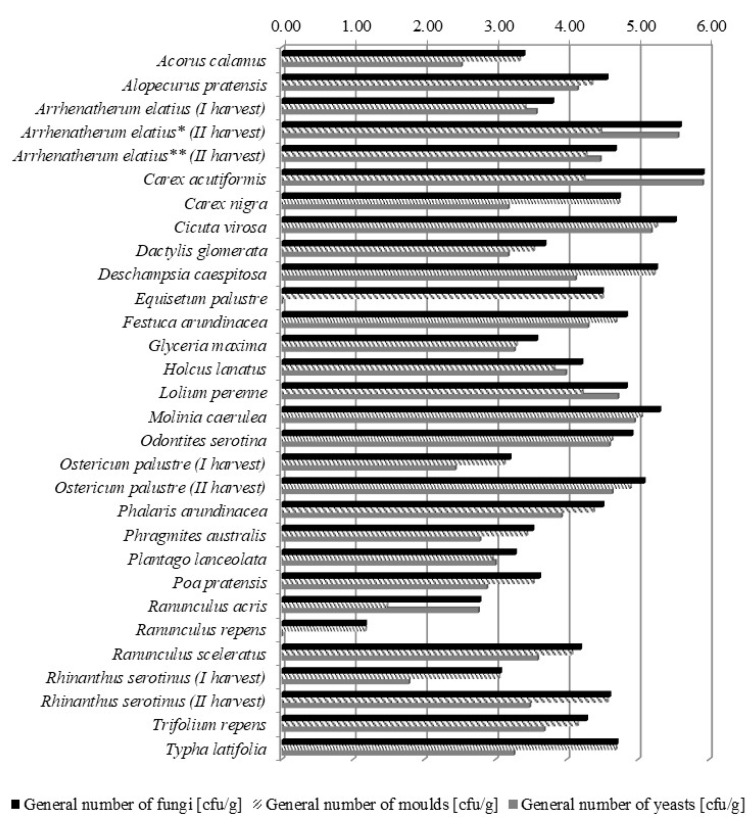
Level of fungal contamination of grass samples. Data are presented using logarithmic scale. * generative phase, ** vegetative phase.

The results of quantitative and qualitative mycological analysis of the meadows are presented in Table 1 and Figure 1. The experimental plot was dominated by moulds belonging to the following genera: *Humicola* spp.—24%, *Alternaria* spp.—20%, *Cladosporium* spp.—18%, *Torula* spp.—8%, *Fusarium* spp.—5%, *Mucor* spp.—5%, *Pythium* spp.—3%, *Acremonium* spp.—1%, *Nigrospora* spp.—1%, *Penicillium* spp.—1%, *Rhizopus* spp.—1%, and unidentified colonies—12%. The remaining 1% was comprised of moulds belonging to the genera: *Arthrinium* spp., *Aspergillus* spp., *Aureobasidium* spp., *Bipolaris* spp., *Botryotrichum* spp., *Chaetomium* spp., *Chrysosporium* spp., *Colletotrichum* spp., *Diplodia* spp., *Drechslera* spp., *Epicoccum* spp., *Morteriella* spp., *Phoma* spp., *Phytophora* spp., *Stemphylium* spp., *Ulocladium* spp.

The highest level of fungal contamination was observed for sedges *Carex acutiformis* Ehrh. (8.5 × 10^5^ cfu/g) (Table 1, Figure 1). The most infested grasses (Poaceae) were *Deschampsia caespitosa* (L.) P.Beauv. (1.8 × 10^5^ cfu/g), *Festuca arundinacea* Schreb. (6.9 × 10^4^ cfu/g) and *Lolium perenne* L. (6.9 × 10^4^ cfu/g). The fungi from genus *Humicola* spp. were prevalent in the *Carex nigra* (L.) Reichard and *Festuca arundinacea* Schreb. samples, whereas *Torula* spp. was the only contamination of *Eqisetum palustre* L. samples (Table 1). The level of fungal pathogens on dicotyledonous plants harvested before the first cut was lower, and on some species such as *Ranunculus acris* L. and *Ranunculus repens* L., almost negligible. The only exception was *Cicuta virosa* L., where the level of fungal contamination was very high (3.4 × 10^5^ cfu/g). All species collected during the second harvest: *Arrhenatherum elatius* (L.) P.Beauv., *Molinia caerulea* (L.) Moench, *Odontites serotina* (Lam.) Rchb., *Ranunculus sceleratus* L., *Rhinanthus serotinus* (Schönh) Oborny, and especially *Ostericum palustre* Besser, should be considered highly infected with moulds.

### 2.2. Cytotoxicity of Meadows

**Figure 2:**
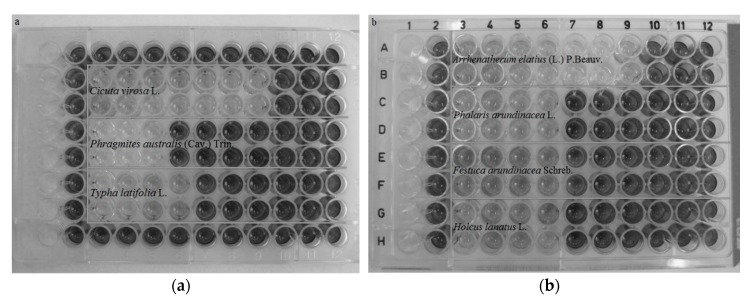
Photographs of the MTT micro-plates with seven extracts containing the plant samples: (**a**) *Cicuta virosa* L., *Phragmites australis* (Cav.) Trin., *Typha latifolia* L.; (**b**) *Arrhenatherum elatius* (L.) P.Beauv., *Phalaris arundinacea* L., *Festuca arundinacea* Schreb, *Holcus lanatus* L.

The MTT test revealed that most of the samples tested (22/30; 73.3%) displayed medium cytotoxicity, whereas 10% (3/30) showed low and 10% high cytotoxicity. No cytotoxicity was found in case of only two Cyperaceae species (6.7%). The highest cytotoxicity at a level harmful to animals was found in *Arrhenatherum elatius* (L.) P.Beauv. (II harvest—vegetative phase) (IC_50_ 1.563 mg/mL) and *Ranunculus repens* L. (IC_50_ 3.125 mg/mL) (Table 1). Other highly cytotoxic samples included *Arrhenatherum elatius* (L.) P.Beauv. (I harvest and II harvest—generative phase), *Cicuta virosa* L., *Plantago lanceolata* L. (IC_50_ 6.25 mg/mL) as well as *Ranunculus acris* L. and *Odontites serotina* (Lam.) Rchb. (IC_50_ 12.5 mg/mL). Based on the MTT test results, both *Carex* species can be considered the least harmful. An example of meadow sward plants cytotoxicity is presented in Figure 2a,b.

### 2.3. Occurrence of Epichloë Endophytes in Grasses

**Figure 3:**
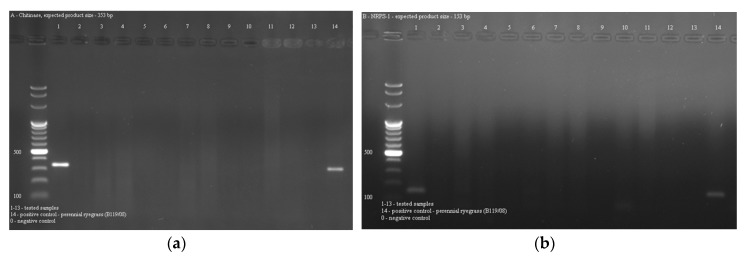
PCR detection of *Epichloë* endophytes in grasses. Gel electrophoresis of PCR products amplified using primers specific to: (**a**) *chitA*; (**b**) *nrps-1* gene. Lane 1: 100 bp DNA ladder; lane 1–13: DNA samples of the examined grasses (1: *Festuca arundinacea* Schreb., 2: *Molinia caerulea* (L.) Moench, 3: *Glyceria maxima* (Hartm.) Holmb, 4: *Deschampsia caespitosa* (L.) P.Beauv., 5: *Alopecurus pratensis* L., 6: *Arrhenatherum elatius* (L.) P.Beauv., 7: *Holcus lanatus* L., 8: *Lolium perenne* L., 9: *Dactylis glomerata* L., 10: *Phalaris arundinacea* L., 11: *Poa pratensis* L., 12: *Carex nigra* Reichard, 13: *Carex acutiformis* L.); lane 14: *Lolium perenne* L. (B119/08) (positive control).

The *Epichloë* endophytes were detected by both pairs of primers in the *Festuca arundinacea* Schreb. sample and in a positive control (Figure 3a,b), but the products amplified for *nrps-1* gene were very weak. That is why PCR assay for *nrps-1* target were additionally optimized by temperature gradient, but with no satisfactory result. The bands visualized in agarose gel were still faint. Thus, *chitA* gene seems to be a better potential indicator for endophyte presence in grass samples.

## 3. Discussion

In the studies undertaken, there were demonstrated differences in the number of fungi and yeasts inhabiting individual plants occurring in the meadow sward. A delayed harvest date can cause the development of many types of moulds that can produce a number of toxic secondary metabolites. At the same time, in the overgrown grasses and herbs, the level of anti-nutritional substances increases, which may affect the assessment of cytotoxicity in the MTT test and deviate from the UVN scales adopted by Klapp [6] and Filipek [5] (Table 1).

Plants that showed high cytotoxicity were not heavily contaminated with moulds. Such high cytotoxicity can be attributed to natural compounds such as cyanogenic glycosides (*Ranunculus acris* L.) and saponins (*Arrhenatherum elatius* (L.) P.Beauv.), which are responsible for the increasing resistance to pests and pathogens (Table 1). However, their presence in hay and haylage could have a negative impact on the rumen microflora. High cytotoxicity of *Arrhenatherum elatius* (L.) P.Beauv. (II harvest—vegetative phase) may result from, in addition to the presence of numerous moulds (4.8 × 10^4^ cfu/g), the presence of saponins. Also the high cytotoxicity of *Ranunculus repens* L. in the MTT test confirmed that it is a plant that contains a toxic component: glycoside—ranunculin, from which anemonine and protoanemonine are released—a volatile substance with an acute odor and burning taste (irritating the skin, mucous membranes of the eyes, nose and larynx), moreover, there are flavonoids: vitexin, neovitexin. Large amounts can cause poisoning and even death of livestock. However, it loses its toxic properties in hay and has a low nutritional value. Also, *Cicuta virosa* L. (IC_50_ 6.25 mg/mL) is a poisonous plant that contains polyunsaturated alcohol—cicutoxin. This substance is also present in hay and silage. This plant is especially poisonous for cattle, horses and sheep [1,3,9,20]. On the other hand, some metabolites can affect the plant beneficially to ensure higher resistance to feeding of many insects and to infection by harmful nematodes without toxicity to animals. Such effect of the endophyte comes mostly from the presence of e.g., peramine, epoxy-janthitrems and lolines. These are the ones which are considered the major elements to condition an increased resistance to various biotic and abiotic stress factors. Peramine and epoxyjanthitrems mostly inhibit the feeding of various pests on infested plants. The lolines, however, are considered to demonstrate a multidirectional effect which decreases e.g., the susceptibility of the plants to infection with pathogens and pest feeding [23,24,25,26]. The presence of endophytes also increases the resistance to drought stress [27]. So, endophyte can serve the plant as Bological Control Agent (BCA) without any detrimental effect for herbivores if the lolitrem and ergot alkaloids are not produced or in the very low level. Some of the identified fungi, e.g., *Trichoderma* spp., *Cladosporium* spp. can be considered as antagonists of many pathogens of grasses and also other plants. Many of *Trichoderma* spp. are known from their aggressiveness to dangerous pathogens e.g., *T. virens* against *Pythium ultimum*, *Rhizoctonia solani*, *Rhizopus oryzae* [28]. Over 60% of commonly used biofungicides are *Trichoderma*-based. Different mechanisms of action of *Trichoderma* spp. are responsible for its antifungal activity, such as hyperparasitism, production of antimicrobial secondary metabolites (e.g., gliotoxin, gliovirin), hydrolytic enzymes, etc. [29]. Recently, symbiotic interactions of *Trichoderma* spp. (e.g., *T. martiale*, *T.* amazonicum, *T. evansii*,) with plants inducing higher resistance of the host to biotic and abiotic stresses have been reported [30,31,32]. Antagonistic properties of *Cladosporium* spp., especially against rust fungi, are also well described in the literature (e.g., *C*. *uredinicola* parasitizing *Puccinia violae*, *P*. *puta* and *Cronartium fusiforme* or C. *tenuissimum* on *Uromyces appendiculatus*, *Cronartium flaccidum*). *Cladosporium tenuissimum* parasiting *Peridermium pini* can act also as biocontrol agent for pine trees) [33]. It is also known that *C. oxysporum* can be used as a biocontrol tool against insect black bean aphid *Aphis fabae* [34]. Those antagonists could be transmitted from meadows to other neighboring plants to ensure higher resistance to different pests.

In Poland, about 70 species of fungi were found on grasses, and 20 species are listed as causing economic damage. The degree of harmfulness of individual pathogens depends on the use of grasses and type of crop [35]. Fungi belong to the natural microflora of the upper soil layer. From an ecological point of view, they play an important role in the natural circulation of matter, through decomposition of biomass. At the same time, as pathogens, they are important in cereal crops and grass habitats, causing a reduction in yields and a decrease in their quality. Through the formation of toxic metabolic products, some of the fungi detected in meadow biomass belong to pathogenic moulds, which along with pathogenic spores, must be included in feed contamination with the greatest harm to health [36]. In particular, these are genera: *Fusarium* spp., *Penicillium* spp., *Alternaria* spp., *Mucor* spp., *Aspergillus* spp. The cited moulds additionally adversely affect the microflora of the gastrointestinal tract, and especially reduce or inhibit the growth of anaerobic fungi in rumen, which reduces the efficiency of macroorganism [7]. In addition, fungi of the genus *Fusarium* spp. may damage the grass at different phases of their growth. They cause seedling fading. They are also the cause of the formation of light spots in the old sod. High temperature promotes the disease, especially when preceded by very humid conditions (e.g., heavy rains). Damaged grasses have brown and black roots and stolons. Sick spots can be a streak, or a ring with healthy plants in the center. Recent studies in America indicate that in some cases such symptoms are caused by soil fungus *Magnaphorte poae* Landsch. & N. Jacks., and fungi of the genus *Fusarium* spp., previously considered a disease factor, colonize damaged plants as secondary pathogens. The author in mycological research has shown frequent grass infestation by moulds of the genus *Pythium* spp., which cause gangrene of grass seedlings [35]. This genus is represented by about 150 species. These organisms are saprotrophs or optional parasites and, as soil organisms, they cause rot in various plant organs, including fruits, seedlings and root diseases of many plant species. Typically, *Pythium* spp. species are polyphagic, i.e., infecting different plant species [37,38]. In the mycological study it was also found that the dominant fungi occurring in the biomass studied were *Humicola* spp. which are cellulose-destroying soil organisms and fungi of the genus *Torula* spp., commonly occurring in nature on withered shoots of various perennials, especially in humid conditions [39].

An important factor for a rational meadow management is the appropriate date for mowing the sward. The introduction of the ‘Natura 2000’ bird directive and the use of meadows in accordance with the agri-environmental programs’ recommendations caused that the first biomass harvest from such areas is delayed, with overgrown and mould-infested vegetation. Such biomass is mainly used for the preparation of haylage, which is then a basic component of TMR. The modern TMR cattle feeding system completely prevents these animals from sorting individual components of the ration. Several years of own studies on TMR samples confirmed that these feeds cause a number of health problems in ruminants [40]. The problem is caused by the inferior hygienic status of haylage, which is the main component of TMR. Earlier feeding with sward grazing and harvesting for hay resulted in the fact that the cattle did not eat the poisonous plant or the low value fodder. By examining the feed from numerous meadow communities and observing the reaction of animals, Klapp [6] and Filipek [5] could qualify individual species of grazing sward to the classes they adopted. However, these were estimation methods for the UVN. In the method, the authors also included general data on the presence of anti-nutritional substances in particular plants. Anti-nutritional substances include, among others, proteolytic and amylolytic enzyme inhibitors, haemagglutinin, glycosides, saponins, alkaloids, phytates or mycotoxins. Most give a bitter taste, resulting in the negative food intake by the animals. Ingestion in larger amounts can lead to disorders in the functioning of an organism and even poisoning [41]. Varied levels of cytotoxicity given in Table 1 can also be indicative of the hitherto less well-known defence reaction of plants against the selective collection of species by animals, a development and sustainability strategy which is partly based on generative growth. Currently, with the appropriate instrumentation equipment, the toxicity of harmful substances can be determined by cytotoxicity tests. The MTT test used in the study indicated the scale of toxicity of individual plants, but did not always correlate with the UVN of sward. Plants well-rated by Klapp [6] and Filipek [5] and willingly chosen by animals, e.g., *Arrhenatherum elatius* (L.) P.Beauv., *Alopecurus pratensis* L., *Dactylis glomerata* L., *Lolium perenne* L. and *Trifolium repens* L. showed high or medium cytotoxicity, while plants of low usefulness value like *Equisetum palustre* L. and *Cyperaceae*, were characterized by low cytotoxicity to animal cells. Furthermore, species such as *Lolium perenne* L. and *Arrhenatherum elatius* (L.) P.Beauv. belonged to a group of the most contaminated grasses. Three plant samples (*Arrhenatherum elatius* (L.) P.Beauv., *Ostericum palustre* Besser and *Rhinanthus serotinus* (Schönh) Oborny) harvested in the second cut showed higher cumber of the total number of fungi as compared to the first cut.

In a study conducted in Poland by Wiewióra and Żurek [16], it was found that endophytic fungi of the genus *Epichloë* most often inhabit the grass species *Lolium perenne* L., *L. multiflorum* Lam., *Festuca pratensis* Huds., *F. arundinacea* Schreb., *F. rubra* L. and *F. ovina* L. The three first species mentioned belong to the most valuable fodder grasses and are also permanent components of almost all sowing mixtures available on the domestic market. In our own studies, the presence of endophytes was found by means of the PCR technique in only one species, *Festuca arundinacea* Schreb.

The undertaken research indicates that in the first cut of meadows within the agri-environmental Natura 2000 program, there are a number of plants in which phytotoxins and mycotoxins can cause a spectrum of clinical symptoms and anatomic-pathological changes in animals. This is confirmed by the large number of fungal spores found. In addition, poisoning may occur due to unidentified phytotoxins. Numerous fungi have been identified, for which there are no recognized toxins (masked mycotoxins). In addition, these compounds may be transferred to edible products of animal origin such as milk and cheese and be a threat for the health of consumers. In the food chain, most plant toxins are biotransformed and therefore the contamination is best assessed in the final product such as milk. In organs and body fluids, it is easier to evaluate mycotoxins and their derivatives, e.g., aflatoxin M_1_ [42,43,44]. There are a number of chemical analytical methods used to determine important phytotoxins and mycotoxins such us (HPLC, GC, GC-MS and LC-MS/MS). The main problems encountered in many of the reviewed methods were the frequent unavailability of suitable internal standards (especially isotope-labelled analogues) and often absent or fragmentary method optimization and validation [45].

Thus, the accepted method of only botanical classification (Klapp/Filipek) of plant material is insufficient. In the near future, a deeper analysis of the fungal infection (species identification by methods of molecular biology, mass spectrometry), plant and mould cytotoxicity (MTT test) as well as a broader assessment of the level of mycotoxin contamination (HPLC-MS/MS) is indicated.

In conclusion, the results of this study may broaden the knowledge of the causes of problems resulting from feeding of roughage (a new system based on TMR), mainly from natural meadows, and help in creating new rankings of the feed value of meadow sward components.

## 4. Materials and Methods

### 4.1. Plant Material Sampling

Plant material was harvested from twice cut meadows located in the valley of the Bydgoszcz Canal. The Bydgoszcz Canal valley, separated as a physico-geographic microregion in the western part of the Toruń Basin mesoregion, is located in the post-flood areas within former pra-Vistula river bed. These areas are used mainly as hay meadows. Currently, they have an area of approx. 4000 ha and in most cases are not fertilized. They are characterized by a multi-species composition with an average utility value due to large degradation of the sward [46,47]. The meadows are included in the ‘Natura 2000’ Bird Special Protection Areas, where a delayed harvest of the first cut is made (in most meadows, since 1 July). Twenty-six plant species (*Acorus calamus* L., *Alopecurus pratensis* L., *Arrhenatherum elatius* (L.) P.Beauv., *Carex acutiformis* Ehrh., *Carex nigra* (L.) Reichard, *Cicuta virosa* L., *Dactylis glomerata* L., *Deschampsia caespitosa* (L.) P.Beauv., *Equisetum palustre* L., *Festuca arundinacea* Schreb., *Glyceria maxima* (Hartm.) Holmb., *Holcus lanatus* L., *Lolium perenne* L., *Molinia caerulea* (L.) Moench, *Odontites serotina* (Lam.) Rchb., *Ostericum palustre* Besser, *Phalaris arundinacea* L., *Phragmites australis* (Cav.) Trin., *Plantago lanceolata* L., *Poa pratensis* L., *Ranunculus acris* L., *Ranunculus repens* L., *Ranunculus sceleratus* L., *Rhinanthus serotinus* (Schönh) Oborny, *Trifolium repens* L., *Typha latifolia* L.) were harvested in 2014 upon reaching the cutting maturity (2–4 plant samples were collected within the designated points, the results of which were averaged). The species included commonly occurring meadow plants from various taxonomic units classified, according to Filipek [5], to various utility value classes, from the best fodder plants (usefulness value number, UVN = 10) to non-fodder plants (UVN between 0 and 3), and even harmful ones (UVN < 0). Most of the plant material for research was collected during June and July. In order to unify the development phases of species with different growth rates, grasses were collected after earing, not later than in the beginning of April, while sedges with fully developed inflorescences and dicotyledons were collected in the flowering stage. The only exceptions were the late phenological species: *Rhinanthus serotinus* (Schönh) Oborny and *Ostericum palustre* Besser that by 30 June did not develop inflorescences and therefore were collected during the vegetative development phase. These two species as well as *Molinia caeruela* (L.) Moench and *Odontites serotina* (Lam.) Rchb., that develop only in the second sward regrowth, were harvested as fully developed, with well-developed inflorescences in the second half of August. In August, samples of *Arrhenatherum elatius* (L.) P.Beauv. were collected again in the generative and vegetative phase in order to verify the earlier results of the MTT test. Samples were collected by a specialist in the field of meadow farming. Immediately after collection, material for analyses was dried in shaded, airy rooms to an average water content of 8–10% (air-dry mass) to limit the development of storage microorganisms and their metabolites. Samples were placed in identical paper bags and stored in room conditions until analysis.

### 4.2. Analysis of the Fungal Contamination

Mycological examination of meadow plant samples was carried out on YGC (yeast extract, glucose and chloramphenicol) agar medium, after 5–7 days incubation at 25 ± 1 °C. The results were expressed as the number of colony forming units (cfu) per gram of a sample. Qualitative and quantitative mycological analysis was carried out according to PN ISO 7954: 1999 [48].

Each sample, weighing about 20 ± 0.2 g, was ground in sterile conditions, suspended in a physiological solution (1:9), consisting of potassium hydrogen phosphate 3.6 g/L, disodium phosphate 7.2 g/L, sodium chloride 4.3 g/L, peptone 1 g/L, pH 7.0 ± 0,2, according to PN-EN ISO 6887-1: 2000 [49] and homogenized in a microbiological homogenizer (Stomacher) for 90 s. Ten-fold dilutions [49] were prepared and surface inoculation was performed according to PN-ISO 7954:1999 [29] on agar plates: YGC (5 g yeast extract, 20 g glucose, 0.1 g chloramphenicol, 15 g agar, pH 6.6) with own modification. Tenfold dilutions were done in triplicate (1 mL of 10 cfu, and 100 μL of 10 to 10^3^ cfu) The general number of moulds and yeasts was determined after 5–7 days of incubation at 25 °C and expressed as cfu/g of material. Initial separation between moulds and yeasts was performed by microscopic analysis.

### 4.3. Identification of Moulds

On the basis of colony morphology and type of sporulation, the dominant genera of fungi were isolated. Identification of moulds genus was performed using selective media and microscopic analysis according to Amann’s procedure (dyes for analysis according to PN-R-64791:1994 [50]). Calculations were performed (cfu/g) for each of the identified genera.

### 4.4. MTT Assay

Cytotoxicity evaluation was performed using the MTT (3-(4,5-dimethylthiazol-2-yl)-2,5-diphenyltetrazolium salt) test, which is a sensitive diagnostic tool used to measure cytotoxic activity of potential medicinal agents and toxic materials. Cells that were not damaged by mycotoxin, can convert a yellow tetrazolium salt, MTT, to a violet, water-insoluble formazan [51]. The reaction takes place in the mitochondria of live cells. The intensity of color reaction is proportional to the number of intact and metabolically active cells and can be measured photometrically. The study was performed with the use of swine kidney cell line (SK) that was derived from a cell culture collection of the Ludwig-Maximilians-Universität München and was kindly provided by Prof. Manfred Gareis, PhD in 2011. The cells were cultured in a medium supplemented with antibiotic solution (penicillin and streptomycin, Sigma Aldrich) and fetal calf serum (Sigma Aldrich), in a CO_2_ Hera Cell incubator (Heraeus, Germany) (5% CO_2_, 37 °C, 98% humidity) [51]. Extracts containing an equivalent of 0.8 g of the plant sample were prepared [52]. The samples were evaporated to dryness in a vacuum evaporator set to 40 °C and the resulting extracts were dissolved in 1 mL of mixture of ethanol dimethyl sulfoxide-minimum essential medium with Earle’s salts (MEM) (1.7 + 0.3 + 98, *v*/*v*/*v*) as described by Hanelt et al. [51]. Then, serial log 2 dilutions of sample extract were prepared (1–400 mg/mL, 2–200 mg/mL, 3–100 mg/mL, 4–50 mg/mL, 5–25 mg/mL, 6–12.5 mg/mL, 7–6.25 mg/mL, 8–3.125 mg/mL, 9–1.563 mg/mL, and 10–0.781 mg/mL). All plates were incubated at 37 °C in a humidified atmosphere with 5% CO_2_ for 48 h. Then, MTT stock solution (20 μL) was added to each well, and plates were incubated for another 4 h. After removing supernatant with a multichannel micropipette, 100 μL DMSO was added to each well, and absorbance was measured spectrophotometrically with an ELISA-Reader. Cytotoxicity was quantified with a micro-plate spectrophotometer (Ledetect 96 Microplate Reader, Labexim Products, Lengau, Austria) coupled with MikroWin 2010 OEM version (Mikrotek Laborsysteme GmbH, Overath, Germany), based on absorbance measured at 510 nm wavelength, which corresponded to maximum absorption of formazan derivative. In the case of absorbance lower than 50% of the cell division activity, all analysed samples were considered toxic. Maximum acceptable toxic levels of each plant sample were estimated as the lowest dilution [mg/mL], at which toxic effect was observed in 50% of the cells (IC_50_, Mean Inhibitory Concentration).

### 4.5. PCR Detection of Endophytes in Grasses

Due to the fact that endophytes are typical for plant species of the Poaceae family, 13 grass species were analysed—*Festuca arundinacea* Schreb., *Molinia caerulea* (L.) Moench, *Glyceria maxima* (Hartm.) Holmb, *Deschampsia caespitosa* (L.) P.Beauv., *Alopecurus pratensis* L., *Arrhenatherum elatius* (L.) P.Beauv., *Holcus lanatus* L., *Lolium perenne* L., *Dactylis glomerata* L., *Phalaris arundinacea* L., *Poa pratensis* L., *Carex nigra* Reichard, *Carex acutiformis* L. As a positive control, sample of perennial ryegrass (B119/08) (*Lolium perenne* L.) known to be inhabited by *Epichloë festucae* var. *lolii*, was used. Dried plant material was homogenized using a Retsch MM400 homogenizer. For each sample, 60 mg of material was collected for DNA isolation. The isolation was performed using a modified Doyle and Doyle [53] technique. The concentration of obtained DNA was measured with a Quantus Fluorometer E6150 according to the manufacturer’s recommendations.

PCR (polymerase chain reaction) amplification was performed according to the procedure of Rasmussen et al. [54] using primers specific to *chitA* gene encoding chitinase A: 5′-AAGTCCAGGCTCGAATTGTG-3′, 5′-TTGAGGTAGCGGTTGTTCTTC-3’ (353 bp) and a primer pair 5′-GTCCGATCATTCCAAGCTCGTT-3′, 5′-TGGTGGGAAGTTCCCTGCAC-3′ (153 bp) specific to *nrps-1* gene encoding nonribosomal peptide synthetase.

## Figures and Tables

**Table 1:** Results of MTT cytotoxicity test and mycological analysis of meadows. The results were obtained for 26 species of grasses included in the classification according to Klapp [6] and Filipek [5]. Literature data on the presence of toxic compounds detected in plant species were also included.

Species	UVN ^a^ According to Filipek [5] from 10 to −3 and Klapp [6] from 8 to −1)	Cytotoxicity IC_50_ (mg/mL) (Classes of Cytotoxicity ^b^)	General Number of Fungi (Moulds and Yeasts) (cfu/g)	Contribution of Moulds Genera (%)	Toxic Factors	
					Factor	Reference
I harvest—June/July						
**Poaceae**						
*Alopecurus pratensis* L.	9 (7)	50 (++)	3.7 × 10^4^ ± 2.3 × 10^3^	*Pythium* (61) *Humicola* (11) *Phytophthora* (11) *Torula* (8) not identified (9)	no data	-
*Arrhenatherum elatius* (L.) P.Beauv.	9 (7)	6.25 (+++)	6.4 × 10^3^ ± 1.3 × 10^3^	*Humicola* (34) *Alternaria* ^c^ (15) *Cladosporium* ^c^ (13) *Ulocladium* (8) *Phoma* (6) *Fusarium* ^c^ (1) *Pythium* (1) not identified (22)	saponins	[9]
*Dactylis glomerata* L.	9 (7)	50 (++)	5.0 × 10^3^ ± 8.6 × 10^2^	*Torula* (46) *Pythium* (16) *Alternaria* ^c^ (11) *Cladosporium* ^c^ (8) *Humicola* (8) *Bipolaris* (2) not identified (9)	endophytes	[13]
*Deschampsia caespitosa* (L.) P.Beauv.	3-0 (3)	50 (++)	1.8 × 10^5^ ± 6.5 × 10^4^	*Humicola* (45) *Alternaria* ^c^ (10) *Fusarium* ^c^ (6) not identified (39)	endophytes	[16]
*Festuca arundinacea* Schreb.	6 (4)	50 (++)	6.9 × 10^4^ ± 9.7 × 10^3^	*Humicola* (82) *Cladosporium* ^c^ (3) *Pythium* (3) *Alternaria* ^c^ (2) *Epicoccum* ^c^ (1) not identified (9)	perlolyrine, cyanogenic glycosides	[9]
					alkaloids produced by endophytes (lolitrem, ergovaline)	[10,13,16]
*Glyceria maxima* (Hartm.) Holmb.	5 (4)	100 (+)	3.8 × 10^3^ ± 8.8 × 10^2^	*Chrysosporium* (48) *Humicola* (21) *Pythium* (20) *Alternaria* ^c^ (3) *Cladosporium* ^c^ (3) *Phytophthora* (2) not identified (3)	cyanogenic glycosides	[9]
*Holcus lanatus* L.	5 (4)	50 (++)	1.6 × 10^4^ ± 4.8 × 10^3^	*Pythium* (27) *Alternaria* ^c^ (23) *Cladosporium* ^c^ (9) *Humicola* (9) *Epicoccum* ^c^ (5) not identified (27)	aconitic acid, cyanogenic glycosides, *Pithomyces chartarum* (fungus)	[9]
					endophytes	[13]
*Lolium perenne* L.	10 (8)	50 (++)	6.9 × 10^4^ ± 9.4 × 10^3^	*Humicola* (45) *Cladosporium* ^c^ (15) *Nigrospora* (5) not identified (35)	perlolyrine, cyanogenic glycosides, alkaloids (mainly lolitrem, ergovaline, peramine), ergot alkaloids -clavines, lysergic acid, lysergic acid amides, ergopeptides produced by endophytes)	[16]
*Phalaris arundinacea* L.	7 (5)	50 (++)	3.2 × 10^4^ ± 4.1 × 10^3^	*Humicola* (56) *Alternaria* ^c^ (16) *Torula* (10) *Phoma* (3) *Fusarium* ^c^ (1) not identified (14)	9 alkaloids including gramine and tryptamine derivatives (N-methyltryptamine), hordein	[9]
*Phragmites australis* (Cav.) Trin.	1 (2)	100 (+)	3.4 × 10^3^ ± 4.0 × 10^2^	*Cladosporium*^c^ (59) *Alternaria* ^c^ (23) *Fusarium* ^c^ (8) *Penicillium* ^c^ (2) *Trichoderma* (1) not identified (7)	no data	-
*Poa pratensis* L.	10 (8)	50 (++)	4.2 × 10^3^ ± 7.9 × 10^2^	*Humicola* (44) *Cladosporium* ^c^ (12) *Pythium* (9) *Alternaria* ^c^ (4) *Diplodia* (4) *Torula* (4) *Mucor* ^c^ (2) *Colletotrichum* (1) *Phytophthora* (1) not identified (19)	endophytes	[16]
**Cyperaceae**						
*Carex acutiformis* Ehrh.	1–0 (1)	nd (−)	8.5 × 10^5^ ± 1.3 × 10^4^	*Pythium* (31) *Humicola* (24) *Fusarium* ^c^ (23) *Alternaria* ^c^ (16) *Cladosporium* ^c^ (3) not identified (3)	flavones (glycoflavon, tricin)	[17]
*Carex nigra* (L.) Reichard	1 (1)	nd (−)	5.5 × 10^4^ ± 9.6 × 10^3^	*Humicola* (84) *Alternaria* ^c^ (3) *Fusarium* ^c^ (2) not identified (11)	flavones (tricin)	[17]
**Other species**						
*Acorus calamus* L.	0 (no data)	50 (++)	2.5 × 10^3^ ± 2.3 × 10^2^	*Alternaria*^c^ (53) *Fusarium* ^c^ (37) *Mucor* ^c^ (4) *Acremonium* (4) *Aspergillus* ^c^ (2)	asarones	[18]
*Cicuta virosa* L.	−3 (−1)	6.25 (++)	3.4 × 10^5^ ± 4.0 × 10^4^	*Cladosporium*^c^ (32) *Alternaria* ^c^ (29) *Mucor* ^c^ (25) *Fusarium* ^c^ (7) *Penicillium* ^c^ (5) *Aspergillus* ^c^ (2)	cicutoxin (aliphatic alcohol)	[19]
					aconitine, benzoaconitine, neopeline, aconine, napeline	[1,20]
*Equisetum palustre* L.	−2 (−1)	100 (+)	3.2 × 10^4^ ± 4.0 × 10^3^	*Torula* (100)	alkaloids (palustrine, palustridine, nicotine), aconitic acid	[17]
*Ostericum palustre* Besser	no data species spreading on two-cut meadows	50 (++)	1.6 × 10^3^ ± 4.6 × 10^2^	*Alternaria*^c^ (20.5) *Humicola* (15) *Phoma* (14) *Chaetomium* (9) *Nigrospora* (9) *Morteriella* (5) *Aspergillus* ^c^ (2) *Botryotrichum* (2) *Aureobasidium* (2) not identified (20.5)	no data	-
*Plantago lanceolata* L.	7–5 (6)	6.25 (++)	1.9 × 10^3^ ± 4.7 × 10^2^	*Alternaria*^c^ (27) *Chaetomium* (17) *Humicola* (17) *Fusarium* ^c^ (10) *Drechslera* (6) *Mucor* ^c^ (6) not identified (17)	iridoid glycoside (aucubin), flavonoids, tannins, organic acids, mucous compounds, pectins	[21]
					iridoid glycosides (aucubin and catalpol), phenolic acids	[17]
*Ranunculus acris* L.	1 (1)	12.5 (++)	6.1 × 10^2^ ± 2.2 × 10^2^	*Alternaria*^c^ (30) *Nigrospora* (30) *Humicola* (20) not identified (20)	glycoside (ranunculin), flavonoids (vitexin and neovitexin)	[17]
					saponins, essential oil, protoanemonin, cyanogenic compounds	[1]
*Ranunculus repens* L.	2 (2)	3.125 (+++)	< 20 ± 1.9 × 10^1^	*Phoma* (40) *Humicola* (40) not identified (20)	glycoside (ranunculin; aglycon: protoanemonin), flavonoids (vitexin and neovitexin)	[1,17]
*Rhinanthus serotinus* (Schönh) Oborny	1 (−1)	25 (++)	1.2 × 10^3^ ± 4.2 × 10^2^	*Alternaria*^c^ (51) *Penicillium* ^c^ (24) *Pythium* (8) *Humicola* (5.5) *Mucor* ^c^ (5.5) *Arthrinium* (3) not identified (3)	aucubin derivatives	[17]
*Trifolium repens* L.	10 (8)	50 (++)	1.9 × 10^4^ ± 7.4 × 10^3^	*Humicola* (28) *Alternaria* ^c^ (11) *Cladosporium* ^c^ (8) *Mucor* ^c^ (6) *Pythium* (4) not identified (43)	tannins, essential oil	[21]
					cyanohydrin glycosides (linamarin) and flavonoids (quercetin and isoquercetin)	[17]
*Typha latifolia* L.	1 (no data)	50 (++)	5.1 × 10^4^ ± 1.1 × 10^4^	*Alternaria*^c^ (91) *Torula* (4) *Fusarium* ^c^ (3) *Cladosporium* ^c^ (2)	no data	-
**II harvest—August**						
**Poaceae**						
*Arrhenatherum elatius* (L.) P.Beauv.—generative phase	9 (7)	6.25 (++)	4.0 × 10^5^ ± 1.1 × 10^5^	*Cladosporium*^c^ (28) *Acremonium* (19) *Alternaria* ^c^ (17) *Fusarium* ^c^ (10) *Pythium* (6) *Mucor* ^c^ (3) not identified (17)	saponins	[9]
*Arrhenatherum elatius* (L.) P.Beauv.—vegetative phase	9 (7)	1.563 (+++)	4.8 × 10^4^ ± 1.3 × 10^4^	*Alternaria*^c^ (26) *Cladosporium* ^c^ (14) *Mucor* ^c^ (13) *Fusarium* ^c^ (6) *Humicola* (5) *Rhizopus* ^c^ (3) *Aspergillus* ^c^ (2) *Penicillium* ^c^ (2) not identified (29)	saponins	[9]
*Molinia caereluea* (L.) Moench	2 (2)	25 (++)	2.0 × 10^5^ ± 4.5 × 10^4^	*Torula* (32) *Alternaria* ^c^ (29) *Cladosporium* ^c^ (18) *Fusarium* ^c^ (9) *Rhizopus* ^c^ (9) *Pythium* (3)	cyanogenic glycosides, ergot	[9]
**Other species**						
*Odontites serotina* (Lam.) Rchb.	1 (−1)	12.5 (++)	8.3 × 10^4^ ± 2.7 × 10^4^	*Humicola* (37) *Cladosporium* ^c^ (33) *Alternaria* ^c^ (15) *Pythium* (3) not identified (12)	iridoid glycosides (aucubin, catalpol), agnuzide	[17]
*Ostericum palustre* Besser	no data	50 (++)	1.2 × 10^5^ ± 1.0 × 10^4^	*Cladosporium*^c^ (80) *Alternaria* ^c^ (8) *Fusarium* ^c^ (8) *Epicoccum* ^c^ (1.5) *Humicola* (1) not identified (1.5)	no data	-
*Ranunculus sceleratus* L.	−1 (no data)	50 (++)	1.5 × 10^4^ ± 1.9 × 10^3^	*Alternaria*^c^ (69) *Fusarium* ^c^ (21) *Mucor* ^c^ (10)	glycoside (ranunculin), protoanemonin and anemonin	[22]
*Rhinanthus serotinus* (Schönh) Oborny	1 (−1)	25 (++)	4.0 × 10^4^ ± 9.8 × 10^3^	*Humicola* (56) *Cladosporium* ^c^ (17) *Alternaria* ^c^ (8) *Stemphylium* (2) *Epicoccum* ^c^ (1) *Mucor* ^c^ (1) not identified (15)	aucubin derivatives	[17]

^a^ UVN—usefulness value number. According to Filipek [5]: 10–9: very good fodder plants; 8–7: good fodder plants; 6–4: medium fodder plants; 3–1: plants of low utility value; 0: plants that do not represent fodder value, are elusive for scythes and are ignored by animals; (−3)–(−1): poisonous plants depending on the degree of toxicity. According to Klapp [6]: 8: fodder crops of full value in all respects; 7–4: significantly useful fodder plants; 3–2: plants with low fodder value; 1: worthless plants; 0: intangible plants when grazing and mowing; (−1): species clearly harmful to animal health. ^b^ Classes of the cytotoxicity: (+++): high cytotoxicity (IC_50_ = 0.781–3.125 mg/mL); (++): medium cytotoxicity (IC_50_ = 6.25–50 mg/mL); (+): low cytotoxicity (IC_50_ = 100–400 mg/mL); (−): no cytotoxicity (IC_50_ > 400 mg/mL); nd: not detected. ^C^ Potential mycotoxin producers.
